# Barriers to Long COVID Care in the U.S.: An Application of Levesque et al.’s Access Framework

**DOI:** 10.1007/s10728-026-00559-0

**Published:** 2026-01-22

**Authors:** Katherine F. Raymond, Twanna Hodge, Beth St. Jean, Brooke Fisher Liu

**Affiliations:** 1 Department of Behavioral and Community Health, School of Public Health, University of Maryland, College Park, College Park, USA; 2 College of Information, University of Maryland, College Park, College Park, USA; 3 Department of Communication, University of Maryland, College Park, College Park, USA

**Keywords:** Long COVID, Health care access issues, Health care navigation, Provider knowledge gaps, Levesque’s conceptual model

## Abstract

Long COVID is a condition that arose during the COVID-19 pandemic in individuals who developed the multi-system chronic condition after a COVID-19 infection. During the pandemic in the United States (U.S.), these “COVID long-haulers” navigated a complex and overburdened health care system in pursuit of diagnoses and treatments. This qualitative secondary analysis used the 2013 Levesque et al. Conceptual Model of Healthcare Access to examine multidimensional health care access issues faced by 29 COVID long-haulers in the U.S. Our analysis showed that long-haulers faced complementary issues from both individual and health systems perspectives related to the inability to get diagnoses or treatments, long waiting times for providers and difficulty reaching services, underinformed providers and biased interpersonal experiences, and struggles with the financial costs of treating the condition, which impacted care decisions. Interviewees also described relying on alternative medicine to provide symptom relief. Overall, this study extends international research by offering a comprehensive examination of Long COVID health care access issues in the U.S. and identifying specific insights related to health care access that made obtaining Long COVID care difficult, such as the mismatch between individual expectations of what health care should look like and how it actually operates. Our use of the full Conceptual Model of Healthcare Access provides new insights into the overlap across layers of access issues and offers suggestions for how public health and clinical health practitioners can collaborate to meet the needs of vulnerable populations such as these in future health emergencies.

**Supplementary Information:**

The online version contains supplementary material available at 10.1007/s10728-026-00559-0.

## Introduction

During the COVID-19 pandemic, public and clinical health infrastructures in the United States struggled to provide adequate health care services for individuals living with Long COVID, a chronic condition resulting from a SARS-CoV-2 infection [1, 4]. Despite millions affected worldwide, knowledge about the health care challenges faced by “COVID long-haulers” and the impact on long-term health outcomes is still emerging [5, 9]. Understanding how individuals navigated health care access for this new chronic condition during a global pandemic is crucial for improving health systems’ responses to future crises [10, 11]. This paper presents findings from a qualitative study of U.S. adults living with Long COVID using the Conceptual Model of Healthcare Access by Levesque et al. [12] to describe the interplay between dimensions of the health system and corresponding abilities of long-haulers when navigating care.

### Background

Long COVID, as it is known in the U.S., develops soon after an individual contracts COVID-19, and presents as fatigue and post-exertional malaise, changes in smell or taste, brain fog, heart palpitations, body soreness, gastrointestinal symptoms, and dizziness, among the over 200 symptoms identified to date [13, 15]. Nearly one in four individuals worldwide and nearly two in ten individuals in the U.S. who contracted COVID-19 developed Long COVID [5, 16]. Access to comprehensive health services for COVID long-haulers varied during the pandemic, with many turning to local public health resources or primary care services for diagnoses, treatments, and medications [17]. Many long-haulers sought longer-term support through newly created Long COVID clinics, which offered initial consultations and referrals with multidisciplinary teams (e.g., physicians, physical therapists, occupational therapists, psychologists) and utilized telehealth care [18, 22].

Despite the availability of various health care services, COVID long-haulers faced similar access issues worldwide primarily due to the newness of the condition [23], the siloing of relevant health specialties [4, 24], competition with the acute care needs of those with COVID-19 [25, 26], the lack of appropriate referrals [4, 23, 27], financial barriers [23, 28, 29], and interpersonal issues with health care providers such as stigma, misdiagnosis, medical gaslighting, etc. [4, 25, 30, 33]. Additionally, some medical providers lacked basic knowledge about Long COVID or did not believe it was a real condition, which impeded care [4, 26, 30]. In the U.S., COVID long-haulers reported difficulties accessing providers, specialty care, and treatments for their symptoms [30].

At a health system level, specialty providers were not always available and primary care providers (PCPs) were unsure to whom they should make referrals [28, 30]. Long-haulers had to be persistent to access the right providers and to have that care covered by insurance, a common issue in other rarer disease patient populations [34, 35]. Individually, long-haulers expected care, support, and answers from their PCPs, but these expectations were often unmet [36]. Additionally, seeking and engaging in care for Long COVID symptoms was emotionally and physically draining for long-haulers, leading to changes in their care-seeking behaviors, such as forgoing care entirely [29, 37]. Those long-haulers who developed Long COVID in the first couple of years of the pandemic had to grapple with a new and exhausting condition during a time of intense social disconnectedness and loneliness which detracted from their ability to engage in self-care behaviors [37, 38]. Even after the pandemic, a lack of awareness of the condition and corresponding social support led to low self-esteem and affected long-haulers’ perceptions of how sick they were [39, 40]. Furthermore, living with Long COVID has economic, quality of life, and mental health impacts on individuals’ lives, which can lead to disability, inability to work or provide for family members, and difficulty contributing to broader societal activities [41, 42]. Overall, these multidimensional individual-level issues shaped long-haulers’ care experiences.

### Theoretical Foundation

**Fig. 1:**
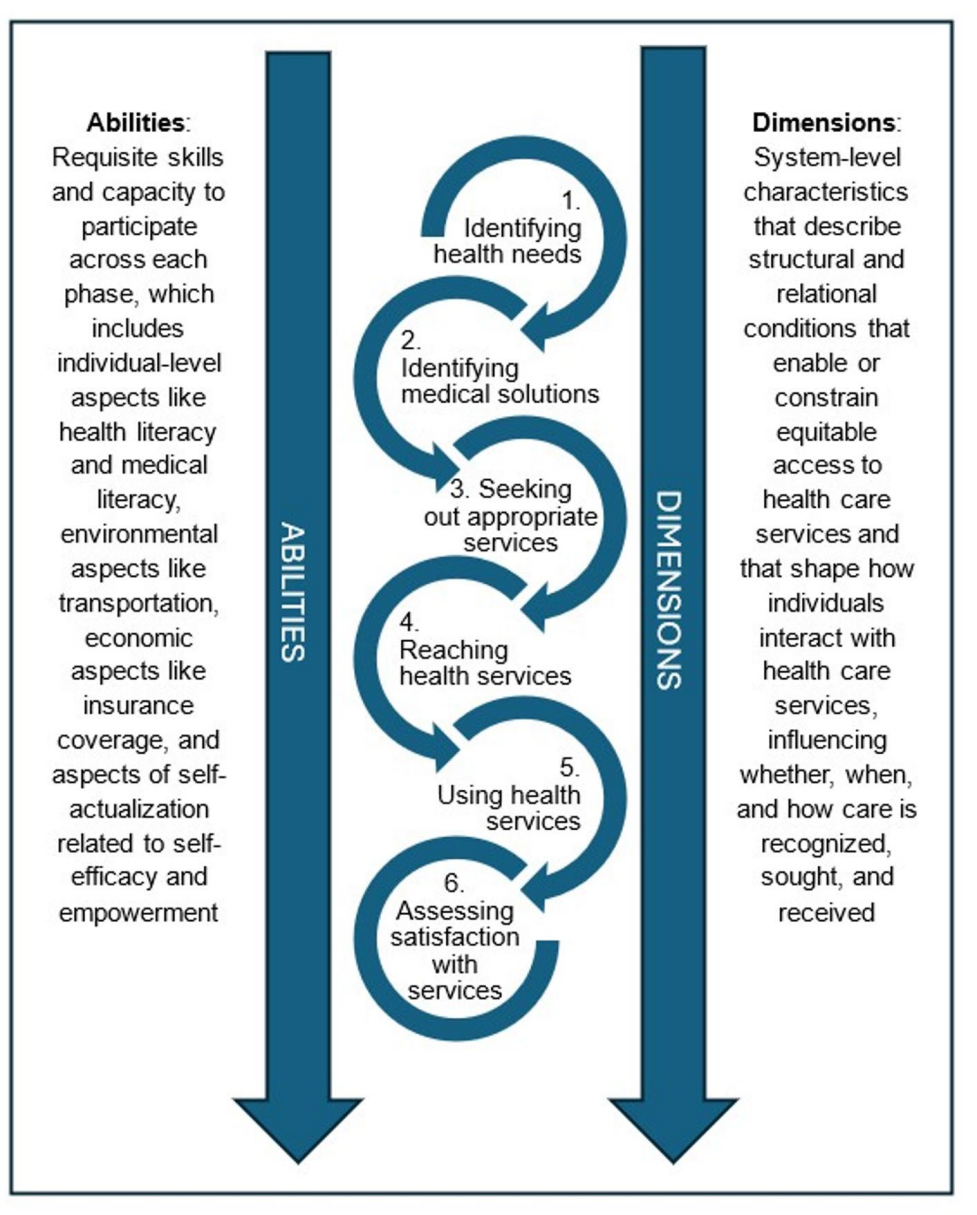
A comprehensive perspective of healthcare access informed by Levesque et al.‘s 2013 model highlighting the multi-step process of health care access

The study of health care access is vast, reflecting the complexity of how access is defined and operationalized. Early research focused on access to services from the provider side [43], which was later replaced by models such as those from Andersen and colleagues [44, 45] and Penchansky and Thomas who sought to describe the interplay between the consumer of health services and the actual services [46]. Later models of health care access account for patients’ perspectives and determinants of health when accessing health services [47]. One such model, Levesque et al.’s “Conceptual Model of Healthcare Access” [12], has been widely used to examine health care access issues from both consumer and system perspectives [48]. The model includes five dimensions: approachability, acceptability, availability and accommodation, affordability, and appropriateness, and five respective patient abilities: ability to perceive, ability to seek, ability to reach, ability to pay, and ability to engage [12]. The model reflects the process of managing chronic conditions in a dynamic context where individual abilities—influenced by demographic, social and economic characteristics—coincide with the characteristics of the surrounding health system (see Fig. 1).

For instance, approachability reflects the presence of a health system with services that can be reached by an individual who perceives that they need care in the first place (1 - identifying health needs and medical solutions). Acceptability refers to cultural and social factors surrounding a health system’s services that impact an individual’s ability to assess if the services they seek are meant for people like them (2 - seeking out appropriate services). Availability and accommodation reflect the timely availability and physical accessibility of relevant health system services, and an ability to reach indicates the physical cost (e.g., time off work, transportation, proximity) of accessing those services (3 - reaching health services). Affordability and ability to pay reflect the bidirectional receipt and expenditure of funds for care (4 - using health services). Finally, appropriateness refers to the quality and effectiveness of care provided by skilled and empathic providers, and is linked closely with an individual’s ability to engage, which refers to one’s health literacy, self-efficacy, and ability to apply medical solutions into behaviors (5 - assessing satisfaction with services).

Many studies partially investigate the dimensions and abilities outlined in the original model, but few examine all 10 domains with long-haulers in the U.S [48, 49]. A Canadian study linked health care access issues among COVID long-haulers to domains like approachability, appropriateness, ability to perceive, ability to seek, and ability to engage [32]. Another study used all 10 domains to explore virtual mental health access for refugees during the COVID-19 pandemic, highlighting the complexities of health care use [50]. An Austrian study found that long-haulers cited barriers and facilitators across most, but not all, of the dimensions and abilities [23]. There is now an opportunity to examine COVID long-haulers’ health care access in the U.S. in a comprehensive way using all 10 dimensions and abilities to understand more about this population’s experience accessing Long COVID care.

### Study Research Questions

This paper presents findings of the health care access issues a sample of COVID long-haulers in the U.S. experienced from 2020 to 2024. Our research questions were:

#### RQ1

What were the system-level barriers related to health care access that individuals living with Long COVID in the U.S. faced during their Long COVID care experience?

#### RQ2

What were the corresponding abilities COVID long-haulers deployed to navigate these barriers?

## Methods

This research study is a qualitative secondary analysis (QSA) [51, 52] using data from a larger multi-method project [76] . The main study, approved by our university’s Institutional Review Board, included data from an online survey (*n* = 135) and a one-time, follow-up, 60-minute semi-structured interview with selected survey respondents (*n* = 29) conducted by four trained interviewers. Survey respondents were recruited across the U.S. through a research-oriented Facebook group for COVID long-haulers as well as through university listservs affiliated with the research team’s schools (e.g., College of Information and School of Public Health). At the close of the survey, respondents could opt in for a follow-up interview to be conducted at a later date over Zoom, and 114 (85%) of survey respondents expressed interest. We invited potential interviewees from diverse backgrounds, with attention to race and gender identity, and enrolled participants in the order that they responded. Recruitment continued among those who expressed interest until we reached data saturation [53, 54] and we received no further replies from interested individuals. Further details on the recruitment procedures can be found elsewhere [77] . Interviews recorded on Zoom were transcribed verbatim using built-in transcription features, reviewed for accuracy, and analyzed using NVivo 14. Interview questions primarily focused on the information needs and experiences of long-haulers, and through the answers to these questions, a range of health care access issues emerged. These data inspired this analysis.

### Thematic Analysis Procedures

We used a thematic analysis approach, modeled after Braun and Clarke [55, 56], to explore health care access issues our interviewees faced. Sample data were drawn from a larger qualitative dataset initially coded by four research team members using 13 inductive codes (comprised of over 200 child codes)(see Online Resources Table 1) and 27 deductive codes. Data tagged with the 13 inductive codes pertained to resources, social or societal factors, affect, beliefs or perceptions, health behaviors, physical & mental health, information seeking or avoidance, information sources, information evaluation, information sharing, information needs, information dissemination, and information use or non-use. These inductive codes served only as an organizing principle and sampling strategy to locate text pertaining to health care access.

To conduct this secondary analysis, the primary author (K.R.) developed a new codebook reflecting the five dimensions and five abilities from the Conceptual Model of Healthcare Access [12] (see Supplemental Table 2 in Online Resources). Data for the analytic sample were then selected in two ways: (1) All coded text falling under the 13 inductive codes (11,315 references across 29 transcripts) was coded again using the new codebook, and (2) We separately searched for the word(s) “healthcare”, “health care”, “doctor”, and “provider” in each participant transcript to capture any missed mentions of health care access issues from the initial coding. Together, these two steps yielded the final analytic sample. The lead author then coded the entire dataset using the 10 access codes followed by axial coding to synthesize the data into emergent themes organized around each pair of dimensions and abilities (e.g., Approachability and Ability to Perceive). To validate the findings, the principal investigator and an additional research team member familiar with the data (B.S. and T.H.) independently reviewed the axial coding data points and resultant themes; discrepancies were resolved through peer-debriefing. The final themes and supporting quotations are presented below.

## Results

### Sample Characteristics

We examined the health care access issues of our 29 interviewees. In our sample (Table 1), 11 individuals first contracted COVID-19 in 2020, seven in 2021, ten in 2022, and one in 2023. More than half (*n* = 18, 62.07%) of interviewees tested positive for COVID-19 once or twice; however, 11 (37.93%) individuals had COVID-19 three or more times. Nearly all interviewees described developing Long COVID symptoms soon after their first suspected COVID-19 diagnosis. Most interviewees (*n* = 26, 89.65%) had private insurance at the time of the interview, with only two individuals reporting public insurance and one individual preferring not to say. Our sample was primarily female (*n* = 24, 82.76%), 23–67 years old (M = 43.1; SD = 13.5), highly educated, and from 17 states across the U.S.

**Table 1:** Participant identifiers, demographics, and long COVID onset

Participant ID^a^	Age	Gender	U.S. state	Highest educational level^b^	Race and/or ethnicity^c^	Date of long COVID onset^d^
P01	29	Nonbinary	California	Graduate	White	05/2020
P02	39	Female	Wyoming	Graduate	White	01/2022
P03	67	Nonbinary	Michigan	Professional Degree	White	05/2020
P04	60	Female	Arizona	Some college	White	09/2022
P05	55	Female	Colorado	Professional Degree	South Asian	04/2021
P06	33	Female	California	Graduate	Asian/Asian American	05/2020
P07	27	Female	Virginia	Graduate	Asian/Asian American	09/2023
P08	23	Nonbinary	Washington	Graduate	American Indian/Alaska Native	06/2022
P09	42	Female	Washington	Professional Degree	White	01/2023
P10	38	Female	Washington	Graduate	White	01/2023
P11	55	Female	Indiana	Graduate	White	11/2022
P12	53	Female	Wisconsin	Graduate	White	02/2022
P13	32	Female	Iowa	Graduate	White	03/2021
P14	29	Female	Virginia	Graduate	Black	11/2021
P15	30	Female	Michigan	Graduate	White	03/2022
P16	53	Female	Michigan	Graduate	White	12/2020
P17	24	Nonbinary	Illinois	Undergraduate	White	07/2022
P18	53	Female	Indiana	Graduate	White	01/2022
P19	63	Female	New York	Graduate	White	09/2020
P20	66	Female	Maryland	Graduate	Mixed race	07/2022
P21	41	Female	North Carolina	Graduate	Hispanic Black	12/2020
P22	40	Female	California	Graduate	White	09/2022
P23	50	Female	Indiana	Graduate	White	06/2022
P24	45	Female	Florida	Graduate	Hispanic White	02/2021
P25	24	Female	New York	Undergraduate	White	01/2023
P26	42	Female	Ohio	Graduate	White	12/2022
P27	63	Female	Illinois	Undergraduate	Black	11/2021
P28	38	Female	Missouri	Graduate	White	09/2020
P29	36	Male	New York	Graduate	White	09/2022

^a^Participant IDs are used to attribute quotations throughout the manuscript

^b^Professional degree includes JD, MD, and others

^c^Non-Hispanic, unless otherwise noted

^d^Date of suspected long COVID onset following a COVID-19 infection

### Thematic Analysis Results

The 2013 Levesque et al. model [12] describes different steps in the health care access process using dyads reflecting the dimensions of the health system and the abilities of the individual interacting with that health system. The following sections and Table 2 feature: definitions based on the original Levesque et al. concepts for each dimension and ability pair, key themes that emerged from the sample of COVID long-haulers, and supporting quotations and ideas from our interviews.

**Table 2:** Terms, definitions, and key themes for dimensions of accessibility & corresponding patient abilities

Access step	Dimensions of health care accessibility		Abilities of COVID long-haulers	
	Definition	Key themes	Definition	Key themes
	**Approachability**		**Ability to perceive**	
I. Identifying health needs and medical solutions	How visible and readily accessible health care services are for the people most in need of those services and who recognize they have a need	Approachability of Long COVID services were largely shaped by timing.	Someone’s ability to recognize the need for care, which is influenced by individual health literacy, knowledge about health, and beliefs related to health and sickness	There was a steep learning curve for patients and providers about what Long COVID is and is not.
	**Acceptability**		**Ability to seek**	
II. Seeking out appropriate services	How much individuals accept aspects of the health care services within the context of surrounding cultural and social factors, beliefs in the systems of medicine, and perceived appropriateness to seek care	Biased healthcare experiences tainted the acceptability of care services.	Refers to concepts of personal autonomy and capacity to choose to seek care, knowledge about health care options and individual rights that would determine expressing the intention to obtain health care	Individuals were navigating the system largely on their own.
	**Availability and accommodation**		**Ability to reach**	
III. Reaching health services	The physical existence of health resources, qualified providers, and varied service delivery modes, that can be reached in a timely manner	Diagnoses opened the door to services, but they were hard to obtain and did not guarantee that qualified care would be available.	How readily mobile an individual is, their access to transportation, their schedule flexibility with work or familial obligations, and their knowledge about health services that facilitates their ability to gain access to a physical health service provider	Individuals cited that adequate local treatments were not available and specialty care was far away.
	**Affordability**		**Ability to pay**	
IV. Using health services	People’s economic capacity to spend resources and time to access, and assume the cost of accessing, relevant health care services	People made decisions based more on expected costs and insurance coverage than expected outcomes.	The capacity to pay for health care services without catastrophic expenditure of resources required for basic necessities	Diagnosing and treating Long COVID was never without cost.
	**Appropriateness**		**Ability to engage**	
V. Assessing satisfaction with services	How well the provided services meet people’s needs related to timeliness, healthcare provider expertise, and accurate assessments and treatments	Care services were not available in a timely way and were unable to offer accurate assessments and treatments for many months to years.	An individual’s participation and involvement in decision-making and treatment decisions, which relates to their capacity to communicate as well as health literacy, self-efficacy and self-management in addition to the importance of receiving care that is actually appropriate for the person, given their resources and skills.	Individuals reported “figuring it out” on their own and finding the need to constantly self-advocate.

#### I. Identifying Health Needs and Medical Solutions (Approachability & Ability to Perceive)

##### Health Systems Level – Approachability

Approachability of Long COVID care services was largely shaped by timing. Someone’s initial COVID-19 experience, including the first signs of Long COVID, and their subsequent treatment and care were very much impacted by when their condition began. Interviewees reported having limited access to diagnostic tools and protective equipment (e.g., PCR or rapid tests, masks, etc.): “*There were no tests. There was no way to officially diagnose this. […] I got it so early in the pandemic”* (P19). Even diagnoses were elusive:*“Long COVID wasn’t even a thing. It wasn’t even talked about… I had never even heard of it; the doctor had never even heard of it”* (P12). They also reported that the health care system was still more focused on COVID-19 care than Long COVID care in those first few years: *“Long COVID kind of kept being relegated… there was a state of urgency in a way*,* so what needed to be tackled first?”* (P24).

Interviewees mentioned how Long COVID did not seem to be a topic on the minds of many care providers who were reportedly not even aware of the condition or how to treat it because they were still struggling to keep up with COVID-19 information while managing burdensome patient loads. Interviewees reported that their primary care staff did not receive training on Long COVID for many years:

I’d go in for checkups […] and people would say, ‘why are you here today?’ And I would say, ‘have you heard of Long COVID?’ And they would say, ‘are you contagious?’ Or they would say, ‘I’ve never heard of that. What is that?’ And it was 2 years before the people in the health care system recognized the phrase (P03).

Overall, many individuals perceived that Long COVID treatments were not visible nor readily accessible. Thus, in those first few years, long-haulers had to refine their ability to perceive what services they needed and how to access them mostly on their own.

##### Individual Ability Level – Ability to Perceive

For ability to perceive, there was a steep learning curve for both patients and providers regarding what Long COVID was and was not. According to interviewees, little was known about Long COVID and symptoms were not well-understood, so many individuals devoted considerable time to figure out what they were experiencing. This uncertainty made it difficult to know how serious it was and what type of care to seek. One interviewee said:

Had I known that in the beginning I wouldn’t have just gone back to work once I felt better, I think I really would have paced myself and kind of taken care of myself and not rushed going back to work. And yet at that time, we didn’t have the understanding of Long COVID. You know, 2020, nobody was really talking about Long COVID (P05).

Whenever their symptoms occurred or re-emerged, individuals reported recognizing that this was a sickness unlike others they had experienced, but they were unsure what it was and how it was treated. Many individuals felt that their PCPs did not know how to diagnose or treat the condition. One individual said: *“[M]y regular doctor is a great doctor*,* but she didn’t really have any answers*,* and it was very slow to get the real diagnosis for Long COVID”* (P25).

Another participant reported:

It seems like it has to get really extreme before medical intervention is offered for a lot of it… Most primary care doctors don’t have a checklist for what to do or what to look for with Long COVID symptoms (P28).

Individuals were often left to navigate their care on their own, especially when they perceived that their providers lacked answers and were not engaged in learning more about Long COVID and available treatments. One interviewee said: “*I did attempt to talk to my doctor*,* but… It wasn’t until more recently that I’ve kind of tried to push to get into these Long COVID-specific clinics”* (P01). As a result, individuals developed their own knowledge about what treatments were supposed to work and who could provide real care. Some individuals reported relying on existing information seeking skills developed from past health care experiences, while others reported that they developed these types of skills due to their Long COVID experiences.

#### II. Seeking Out Appropriate Services (Acceptability & Ability to Seek)

##### Health Systems Level – Acceptability

Biased health care experiences tainted the acceptability of care services for long-haulers. For example, past and current discriminatory experiences (e.g., sexism, ableism, racism, mental health stigma, medical gaslighting, fatphobia, and ageism) with specific types of providers based on aspects of an individual’s identity or health status led individuals to feel discouraged when seeking care and when engaging with certain providers. One interviewee recalled:

I remember still, when I was starting to see specialists, some specialists weren’t very well-versed in it at all, and it’s because it primarily affects women… They just thought it was like, ‘oh, lady people crazy disease’, you know, ‘they’re just… whining and complaining’… (P01).

Additionally, interviewees reported that when they became ill with Long COVID, primary care providers were often skeptical and unhelpful. This led to negative interactions, as described by one interviewee: *“The doctors I talked to were bad and I am uncertain when that will get any better”* (P17). Another individual recounted:

[M]y doctor has been kind of oh, I don’t know, a little unsympathetic, a little frustrated because I’m not improving and he didn’t think I had Long COVID. […] He didn’t think Long COVID was a real thing until I presented to him. So, that really hurt (P10).

Interviewees also described their Long COVID care experience as political due to the surrounding political environment: *“The political voice kinda drowned the medical knowledge*,* and it became very noisy. So*,* I just stayed away and minded the advice that I got from people I trusted”* (P16). When providers sometimes brought their politics into conversations, interviewees reported feeling frustrated:

The primary care physician who I was seeing during the worst of the COVID … he said, ‘Oh, I don’t really believe Long COVID is a thing, I think the media is overblowing it’ and then I would send him … something from the Lancet or from Cell or from Nature about Long COVID … mirroring the symptoms I have … and then he’d be like ‘oh, okay’ (P10).

These interactions early in the relationship influenced how acceptable interviewees found available health services, with one individual reporting:

I think…there are doctors here in the state that I live in who sort of don’t believe in COVID at all, let alone Long COVID. And I feel like it’s that first interaction with your doctor that is so important in terms of how you…respond to your positive test and the ways in which you can try to minimize the chances of you getting Long COVID (P11).

Overall, cultural factors regarding the acceptability of COVID-19 and Long COVID as serious health concerns, coupled with interpersonal factors that led individuals to perceive that healthcare providers were biased against them, negatively impacted long-haulers’ experiences.

##### Individual Ability Level – Ability to Seek

One key theme related to the ability to seek care was that individuals were navigating a disjointed, opaque, and siloed health care system on their own. These conditions impacted the effectiveness of their searches for appropriate primary care and specialty care providers. For instance, one interviewee said: *“I had to piece together information from so many different sources […] [It] would have been nice if it wasn’t so hard to find answers”* (P25).

Another participant discussed the personal toll it took to engage in care seeking in this capacity:

But because I know that the system’s broken and that everyone, all the health care providers…they have great intentions, but they are burned out. They don’t have the resources to do what they need to do, and everything is so siloed… I know I have to, if I want to get better, I have to be persistent and I know it takes a lot of time, energy and stresses me out (P09).

Interviewees reported feeling hopeless or like their attempts to seek out care and treatment were at times futile since they did not know how to most effectively seek out care for symptom relief. One interviewee said,

I’m sure if a new thing were to come up for me … I would get on some wait list again. And I think that is really the barrier, because there just isn’t the resources and support in that way, which I know is true, for so much of the health care system (P02).

Several interviewees described using their personal autonomy to ultimately opt out of traditional care and opt into alternative sources of medicine through non-traditional care providers like naturopaths or acupuncturists, which often resulted in more satisfying health outcomes. For example, one interviewee said:

I saw a naturopath […]. [S]he’s the only doctor that listened to me and honestly knows the most about COVID, instead of any traditional doctor I’ve seen. Because she’s not trying to just treat the symptoms. She’s trying to treat the root cause of inflammation (P08).

#### III. Reaching Health Services (Availability and Accommodation & Ability to Reach)

##### Health Systems Level – Availability and Accommodation

For availability and accommodation, COVID long-haulers faced many barriers when attempting to access Long COVID-specific services, such as diagnoses or specialty care, that were necessary to help them manage their condition. For example, interviewees reported that having a Long COVID diagnosis was critical to facilitating access to follow-up diagnostic tests, clinical interventions, care planning, and disability services. Many of our interviewees, however, contracted COVID-19 early in the pandemic and were unable to “prove” they had Long COVID. This difficulty in “proving” Long COVID in a timely way, according to interviewees, was due to the lack of available tests, patients’ inability to be seen in-person by doctors, and the lack of established policies and procedures designated to identify Long COVID. As one interviewee who contracted COVID-19 early on said, *“The diagnostic tests weren’t available. I mean*,* they did have some diagnostic tests*,* but they weren’t readily available and there weren’t enough of them”* (P03). Another interviewee shared the consequences of this,

I guess there’s no way that really tests for it, at least unless there is and I’m not aware of it. That’s definitely decreased my interest in trying … to figure out exactly if that is what it is and to take action (P22).

Additionally, there was no clear standard of care or treatment plan for long-haulers. Interviewees described that primary care providers and their staff lacked knowledge on who to refer patients to and did not help patients coordinate care:

I had a problem when my medical professional could not be as transparent with me to tell me she wasn’t sure what she needed to do to move forward, to figure out how to work through this…And so it wasn’t until this past fall that I ended up getting the medical help that I actually did. But that was 3 years after the fact (P21).

Furthermore, the availability of access to Long COVID clinics was scarce, difficult to navigate, and often unproductive. Individuals reported having to travel long distances to reach specialty care and were still met with inadequate care experiences due to overburdened providers, long waiting times for appointments, lack of in-person care options, and intermittent access to information about treatments and care. One participant recounted how they used their professional network to access care at a Long COVID clinic after being initially turned away:

I’m on a research grant with… the director of the Long COVID clinic. … And she’s like, ‘Oh no, we would love to have you at our clinic. This is what you do.’ … Her people still turned me away 3 times until I name dropped her. And I said… ‘Do you want me to screen share the email from your boss saying that I should come to the clinic?’ ‘Okay, fine, we’ll make you an appointment’ (P03).

##### Individual Ability Level – Ability to Reach

A key theme individuals discussed for ability to reach was that adequate local treatments were not available, and specialty care was far away. Interviewees explained that because PCPs were ill-informed on the needs of Long COVID patients, they had no way to treat their condition: “*[W]hat treatments are there out there for me? I’m not expecting it to be immediately cured. But what is out there that I have access to*,* specifically*,* that can help me get better?”* (P09).

Individuals also reported that because PCPs were unfamiliar with the Long COVID clinic structure and how to navigate the supporting care network of Long COVID care providers, interviewees could not get the right referrals. One participant remarked,

The health care thing is a journey to get to… the amount of times you’re on a list, or even getting on the Long COVID list and then getting the referral to the Long COVID clinic. It was months before I was seen (P02).

Additionally, interviewees considered vaccines and Paxlovid as important tools to prevent re-infections with COVID-19 and exacerbations of Long COVID symptoms, yet their ability to secure these tools diminished when federal funding ended. An interviewee said,

I did the [Home Test to Treat] program and they gave me Paxlovid, but they mailed it to me after the five-day period of being able to take it. So now I just have it because I can’t take it. And I’m like God forbid I get a tenth infection (P08).

Lastly, specialty care in the form of available providers, specialists, or Long COVID clinics were not available in every town or city. Individuals were forced to either delay care, reject it, or find a way to travel great distances to access the care.

My doctor in the Long COVID clinic had a lot of practical suggestions for that as well. So that was helpful. OK, nine month waiting list wasn’t super helpful but. … And you know it’s a 5-hour drive too (P13).

Our interviewees all reported having difficulty reaching treatments, providers, or physical clinics due to geographic distance, issues with transportation due to their Long COVID symptoms, or uncertainty related to where to go and how to get in the door of certain providers.

#### IV. Using Health Services (Affordability & Ability to Pay)

##### Health Systems Level – Affordability

Regarding affordability, interviewees often weighed medical decisions based on costs, insurance coverage, and the value of clinical outcomes. Some long-haulers reported forgoing expensive diagnostic tests or other out-of-pocket expenses because the expected benefit of those results or treatments did not always clearly outweigh the financial costs:

There’s this… fear of am I going to spend thousands of dollars for people to tell me ‘Yeah, we still don’t know.’ […] Unfortunately, I’m a grad student. My budget is very limited… I gotta choose and I’m not going to be spending thousands of dollars for you to tell me that you still don’t know (P24).

Despite interviewees reporting some medical literacy, navigating care took a physical toll on them. They expressed hope that their health care providers could help them with the emotional cost of care navigation by connecting them to subsidized care or referring them to clinical studies:

I asked her if… she knew people who were actually conducting studies that I could join that would help kind of cover the cost of the all those tests that she wanted me to get done. … She didn’t know anything (P05).

Not every interviewee mentioned affordability concerns or issues related to ability to pay; however, most of the interviewees indicated that they had to consider the economic and emotional costs of engaging in health care for their Long COVID symptoms. Many participants mentioned the importance of having employer-sponsored health insurance cover necessary treatments and how the threat of job loss impacting that insurance coverage was a source of stress. One interviewee shared their struggle: *“Right now I’m on medical leave again and I was going to try to apply for disability. But I’ve been unsuccessful so far and it’s because I can’t prove that I have [Long COVID]”* (P11).

##### Individual Ability Level – Ability to Pay

A key theme regarding ability to pay was that even the basics of diagnosing and treating Long COVID required the expenditure of time, money, and energy. As one individual said, *“I had to just show up to an immunologist’s office at the university because…I was staff and I was on the plan …I didn’t even know if I was going to get any of it covered*,* given… I didn’t get a real referral”* (P17).

Once individuals were able to get Long COVID diagnoses and referrals to the appropriate providers, they had to make decisions about what price they were willing to pay for key services, what medications or treatments they were willing to do without, and what the running total was of their Long COVID costs. They had to know when to make tradeoffs due to high-deductible insurance and the residual financial burden services would yield:

[T]hey were recommending all these tests for me because I was having a lot of heart rate fluctuations and trouble breathing. But they were so expensive, I had to make the decision as to whether to get those tests. I still don’t know if I have cardiac damage because I never completed all that testing (P05).

Individuals represented a spectrum of being willing to pay extreme amounts for any type of support to feeling like they could live with discomfort or mild pain to avoid large out-of-pocket costs. One individual said: *“I had to pay out my ass to get a psychiatrist and a therapist to manage everything…I had access to all these doctors*,* and I would just… front the money…our health insurance sucked”* (P06).

On top of managing the day-to-day symptoms of Long COVID, like brain fog and disorientation, individuals also had to manage the logistics of navigating Long COVID care, such as assessing what services would be covered, what medications were going to cost, and how to navigate insurance coverage for those necessary services. As one participant said,

I go to the pharmacy a lot more often than I used to, so having health insurance and pharmaceutical coverage prescription coverage is more important than it used to be. It is a source of anxiety that I have to manage (P29).

Many interviewees mentioned a sense of desperation for answers and relief that they went out of their way to use alternative care options, like massage therapy, Reiki, and other complementary medicines, to support their health, knowing that these would lead to additional economic costs.

#### V. Assessing Satisfaction with Services (Appropriateness & Ability to Engage)

##### Health Systems Level – Appropriateness

The overarching theme on appropriateness was that clinical care services available for long-haulers were lacking in quality and timeliness. Many interviewees deemed their Long COVID care as largely inappropriate due to the lack of evidence-based services, potential and actual harm from incorrect treatments, lack of access to qualified providers, and inaccurate assessments. For instance, interviewees reported that early COVID-19 testing did not always yield answers or was not sensitive enough to detect nuances in chronic conditions. One participant described their frustration:

I expressed my concerns, and the doctor was like ‘your results look fine, but I don’t know why you’re still experiencing this…I can only tell you that you don’t have heart-related disease. But it can be the COVID really long-lasting symptoms, and I can’t do anything about that’ (P07).

Interviewees described how providers initially thought that their Long COVID symptoms were due to weakness of specific organ systems and recommended (now contraindicated) therapies, like cardiac rehabilitation. One individual reported suffering after being told to go to physical therapy by Long COVID clinic providers:

[A]nd what they had me doing was cardiac rehab … and it made me worse. So the medical profession, their number one priority, is to not cause harm, right?… That’s the first part of the Hippocratic oath. And they absolutely caused harm, 100% lowered my baseline permanently with physical therapy that was not tailored to Long COVID (P12).

Similarly, another interviewee was told, *“’[O*]*h you just need to exercise it out’ and I’m like really? Not it”* (P23).

Many participants described a health care system that was unhelpful and focused more on preventing exacerbations or declines in conditions than curing anything. One interviewee shared,

The most that my doctors have been able to do is, ‘Okay, if you’re having issues with your lungs, maybe some physical therapy for your lungs’. … It’s just more so just monitoring to see if everything’s getting worse [or if] things [are] getting better (P14).

Another individual mentioned the limits of their provider: *“My … specialist … he’s been helpful in terms of diagnostics to figure out exactly what [in] my body is going wrong*,* but hasn’t really provided a ton of great information for me”* (P26).

Individuals were unable to see providers with the appropriate expertise to treat their Long COVID symptoms or offer person-centered care, leading to a loss of confidence in the provider’s abilities. For example, one participant reported:

I was going to get blood work done and I tell the tech that I have Long COVID, she said, ‘What’s that?’ And this was in March of last year [2023]. I was like, ‘Who are you and why are you… taking blood out of my body?’ (P11).

When doctors only treated symptoms, interviewees felt unsupported and mistreated. Additionally, interpersonal mismatches between provider skills and patient needs within a complex, non-patient-centered environment led to haphazard care, discrimination, and distrust in the medical institution.

I thought my primary care doctor and psychiatrist should have been on the same page. My primary care doctor didn’t actually seem to believe in the vaccines. It was like, ‘Oh, we don’t need them no more. Oh you don’t need a mask. Oh, let me be the doctor…’ and he was a really good doctor, but no, even telling him my psychiatrist did the test and says I have long term COVID…It didn’t seem to make a difference (P27).

A different interviewee felt “pre-dismissed” because the providers did not help:

I felt pre-dismissed, if anything. ‘Can I get some Codeine for this bronchitis?’ No, they’re not gonna give me that. … ‘Is there anything else on the planet that will help with my symptoms?’ Apparently not, you know, so I didn’t reach out for any assistance (P20).

Interviewees reported that if providers were able to look at them holistically, and in a patient-centered way, they felt they were more likely to get connected to the appropriate services.

##### Individual Ability Level – Ability to Engage

Regarding the ability to engage effectively in their care experience, individuals reported using all of their skills to figure out how to continually self-advocate to access the care they felt was most appropriate for their symptoms. For instance, one interviewee expressed their frustration with doctors who were missing their concerns:

I think one doctor said there’s not going to be a magic pill that fixes everything and… I never said there would be. … And the neurologist was like, ‘Oh, you don’t have X, Y, Z, but you know, sugar diabetes can cause neuropathy’ and I’m not diabetic… (P15).

All interviewees reported that they built Long COVID self-efficacy and health literacy skills to navigate their care experience. One interviewee said, *“I took the scientific literacy I learned in my education and applied that to learning how to interpret medical studies*,* so that I could be a more involved participant in my own health care”* (P01).

Individuals also reported developing their own care homes and leading their own care teams to fill a need for coordinated care and services. For instance, one individual found a physical therapist on their own who was able to provide a lot of help: *“I found a physical therapist … whose bent was neuro … cognitive things. And she took me on. … I had a lot of relief there”* (P18).

In the end, interviewees described becoming experts in their own care as they realized that they were largely on their own when it came to a longer-term treatment plan and symptom relief:

I probably went to a total of eight different doctors and was in the hospital and saw three or four more. …None of them wanted to look at Long COVID as the potential problem. … So as my symptoms evolved and have changed, and once in a while rear their ugly head even now, I am the one that feels like I’m in control to go solve that. Not necessarily…relying on our health care system (P04).

All interviewees indicated that they had to engage in learning how to navigate health care and deploy these different skills to overcome constant hurdles related to insufficiently trained providers, inadequate care services, unavailable care services, and inappropriate treatments.

## Discussion

Our thematic analysis of 29 interview transcripts with COVID long-haulers showed that they experienced barriers to health care access across all five dimensions and corresponding abilities emphasized by Levesque and colleagues. There was some overlap between the dimensions and abilities within pairs, such as “affordability” and “ability to pay”, and across dimensions, such as “appropriateness” and “availability and accommodation”. This type of overlap is often expected when using this model due to the multidimensionality of health care access and the interconnectedness with social determinants of health [12, 48].

To answer our first research question (RQ1), we found that individuals primarily faced system-level barriers related to the unavailability of diagnostic tools and appropriate treatments, ill-informed and biased health care providers, disjointed Long COVID care systems, and expensive health care services. Our findings align with similar Long COVID research that identified high costs, unavailable clinical staff, long waiting times, transportation issues, and health insurance coverage gaps as primary causes of unmet health care needs [23, 29]. Notably, the presence and impact of provider biases and whether or not they considered Long COVID to be a legitimate condition influenced the acceptability of health care services because feeling believed and validated mattered, while being dismissed eroded trust and confidence in the medical system [26, 57]. Similar sentiments were echoed by individuals in the U.K. [31]. and Greece [58], who reported encountering health care professionals who did not believe patients and were underinformed about Long COVID, which led to feelings of distrust in the health care system [59]. Unique to our study, interviewees perceived a politicization of health services and described its impact on the type of care they received, a finding that merits attention as it suggests that quality of future care might likewise be swayed by politics and medical mis/disinformation [60, 62].

Regarding our second research question (RQ2), we also found that interviewees described an inability to perceive what was really happening within their bodies, an inability to reach key health services in a timely manner, and the inability to find care providers who could support them on every step of their Long COVID health care journey. Skilled, specialized care for the wide-ranging condition was limited nationwide in those first few years of the pandemic [63], although more care options emerged over time [23, 26]. Individuals reported having to piece together their own care homes, develop their own care plans, and treat themselves at home. Perhaps unsurprisingly, interviewees also reported that they based care decisions on whether or not those services would be covered by insurance, a reasonable decision given the high costs associated with Long COVID care [64, 66]. These sentiments reflect the grim landscape of health services in the U.S. and many countries worldwide where individuals experience physical and mental health consequences because they cannot truly afford care.

The health response to Long COVID during the COVID-19 pandemic reflected the space between biomedical and public health realms, acute and chronic care [67]. A variety of care models were developed and deployed in care settings to support individuals with Long COVID in the U.S [19]., which may have contributed to a feeling of disjointedness and mismatched care expectations. A novel insight our interviewees described pertained to an expectation that the health care system they were encountering should have been well-organized and interdisciplinary, yet that is not how the U.S. health care system operates [68]. Part of this mismatch might also have been due to individuals’ desire for symptom alleviation, or palliative care, instead of traditional medicine. Our interviewees reported turning to complementary and alternative medicine (CAM), such as acupuncture, supplements, or chiropractic care, to relieve Long COVID symptoms, which has been studied in long-haulers in the U.K. [69]. Despite a wariness about the pseudoscience of CAM, interviewees described turning to these services because they were readily available, relatively affordable, easier to navigate, and more effective at symptom relief, which aligns with other research exploring the acceptability of and engagement in this type of health service [70, 72].

### Limitations

Our study had some important limitations. First, the convenience sampling approach of our study led to a study population of primarily white, female, and highly educated interviewees with private insurance. Additionally, four (14%) of our interviewees identified as nonbinary, surpassing what might be expected by chance [73]. While Long COVID is much more prevalent and impactful in female and gender minority populations, which likely influenced who was interested in our study [74, 75], future research on this topic could employ probability-based sampling methods to identify and recruit a more representative sample to ensure that the full range of Long COVID experiences are well-represented across demographic groups. Second, a key limitation of this study is the reliance on a single coder for the thematic analysis. While this approach ensured a high degree of consistency, it introduced potential for researcher bias and may limit the generalizability of the findings. To mitigate this potential bias, two others conducted spot checks of the coded and thematically organized data to verify the consistency of the findings. Finally, this study was designed as a secondary analysis which limited the depth and breadth of findings related to health care access. Future research could more comprehensively apply this conceptual model to elucidate the complex interplay of factors influencing health care access for individuals with Long COVID while also measuring the health system access issues identified.

### Implications

Using Levesque et al.’s Conceptual Model of Healthcare Access [12], we described the interlocking and overlapping health care access issues COVID long-haulers faced, expanding existing research using this model with deeper insights into the complexities that contributed to an opaque, unsupportive, and unsatisfying patient experience. Findings from our study highlight an opportunity for health administrators, providers, and professionals to identify how the public health infrastructure and health systems can better collaborate to ensure that emerging health threats such as Long COVID can be identified as early as possible, providers can more quickly build new scientific knowledge [3], and complementary and alternative medicine, when appropriate, can be used to help fill health service gaps. This study’s findings should inspire researchers and practitioners to consider how society will address the health services needs of individuals experiencing novel and evolving health threats during future crisis events.

## Supplementary Information

Below is the link to the electronic supplementary material.

Supplementary material 1 (PDF 253.5 kb)

## Data Availability

No datasets were generated or analysed during the current study.
