# Supplementary material for: Barriers to Long COVID Care in the U.S.: An Application of Levesque et al.’s Access Framework
Source: Health Care Anal. 2026 Jan 22;34(2):162–86. doi: 10.1007/s10728-026-00559-0 (PMC13212712; doi:10.1007/s10728-026-00559-0)
Supplement: Supplementary file 1 — Supplementary material 1 (PDF 253.5 kb) [file 10728_2026_559_MOESM1_ESM.pdf]

Online Resources:

Barriers to Long COVID Care in the U.S.: An Application of Levesque et al.'s Access Framework

Health Care Analysis

Katherine F. Raymond, MSW[a]*, Twanna Hodge, MLIS[b], Beth St. Jean, PhD[b], Brooke F. Liu, PhD[c]
[a]University of Maryland Department of Behavioral and Community Health, School of Public Health, College Park, MD, United States
[b]University of Maryland College of Information, College Park, MD, United States
[c]University of Maryland Department of Communication, College Park, MD, United States

*Corresponding author: Katherine F. Raymond, raymondk@umd.edu, University of Maryland School of Public Health, College Park (MD), United States. ORCID: 0000-0003-2872-8709

**Supplemental Table 1: Original child and parent codes from larger research study.** Parent codes aggregated coding from child codes in NVivo, and these data comprised this qualitative secondary analysis dataset.	
**Parent Code**	**Child Code**
**Affect**	Anger
	Anxiety, Stress, or Worry
	Change in affect or outlook
	Depression
	Disappointment
	Discouragement or discouraged
	Embarrassment or Shame
	Emotionality
	Empowerment
	Encouragement
	Fatalism, Giving up, or Resignation
	Fear
	Feeling safe or unsafe
	Flourishing or Thriving
	Frustration
	Grateful
	Grief, Grieving, Loss
	Hopefulness
	Hopelessness
	Lonely
	Lucky or Fortunate
	Optimism
	Pessimism
	Pride
	Relief
	Resilience
	Self-pity
	Surprise
	Trauma
	Upset
	Vulnerable

**Beliefs or Perceptions**	Ability or inability to understand
	Acceptance, Adaptation, or Integration
	Awareness
	Causal attributions
	Certainty or Uncertainty (management)
	Cognitive limitations
	Confidence or Lack of confidence
	Confirmation bias or Filter bubbles
	Confusion
	Control or Autonomy (or lack thereof)
	Coping or Strategies for managing long COVID
	Curiosity
	Denial
	Desire for future country or govt response to pandemic / How to improve information-related experiences
	Flexibility
	Goals or Goal setting
	Health-related self-efficacy
	Help that didn't help or Missed opportunities to help
	Impacts of (long) COVID
	Inadequacy of country or govt response to COVID
	Information overload or feeling overwhelmed by info
	Information-related self-efficacy
	Knowledgeable or self-assessed expertise
	Openness to information
	Priorities or Prioritization
	Religion or Faith
	Rules or rule-bending
	Self-Reliance or feeling it's up to me
	Strange or Weird
	Success or Failure
	Wishes or Fantasies
**Health Behaviors**	Health behavior change
	Health behaviors

	Perceived severity
	Perceived susceptibility
	Preparing for future COVID outbreaks or other pandemics
	Protective behaviors
	Risk perception
**Information Dissemination**	Cues to action
	Information providers
	Information tailoring
	Outreach
	Public health messaging
	Raising awareness or Educating
	Research funding
**Information Evaluation**	Biased or Unbiased information
	Contradictory (or matching) information
	Information evaluation
	Literacy or Critical thinking skills
	Misinformation or Disinformation
	Perceived accuracy
	Perceived credibility or trustworthiness
	Perceived expertise
	Perceived relevance
**Information Needs**	Changes in information needs
	Desired information, type of information, ideal info resource
	Information needs or questions
	Not being able to put information need into words
	Not being aware one has an info need
	Receiving or finding information too late
	Sufficient or insufficient information
	Unanswerable questions
	Unmet information needs or unanswered questions
**Information Seeking or Avoidance**	Active information avoidance
	Active information seeking
	Barriers to information seeking

	Changes in information seeking over time
	Cross-verification or Use of multiple sources
	Doing my own research
	Factors demotivating information seeking
	Factors motivating information seeking
	Future plans to look up or find information
	Information encountering or Serendipity
	Information orientation
	Information-related preferences
	Joint information seeking with someone else
	Monitoring or Keeping up to date
	Not seeking information
	Passive information avoidance
	Passive information seeking
	Proxy information seeking for someone else
**Information Sharing**	Active information sharing
	Advice
	Changes in information sharing
	Passive information sharing
	Purpose of information sharing
	Reasons for not sharing information
	Reasons for sharing information
	Receipt of shared information
	Recommended information resources
	Target Audience
	Vetting
**Information Sources**	Accessibility or Availability of information
	Celebrities or Public figures
	Changes in information sources consulted or opinions toward info sources
	Doctors or Healthcare providers
	Government agencies
	Internet
	Learning from one's own body or experiences
	Learning from others' experiences or stories

	Most or least helpful information sources
	Most or Least influential information source
	Most or least trusted information sources
	Referrals to specialists or long COVID clinics
	Support groups or Online communities
	Views toward particular information source
**Information Use or Non-Use**	Barriers to information use
	Changes in willingness or ability to use information
	Ease of use or Actionability
	Factors demotivating information use
	Factors motivating information use
	Information management
	Information non-use
	Information use
	Learning or Changes in knowledge
	Reasons for not using information
**Physical & Mental Health**	Change in Mental health or Well-being over time
	Changes in physical health over time
	Delayed diagnosis
	Diagnosis
	Disabled, Incapacitated, or Debilitated
	Disruption, Interruption, Discontinuity in life caused by (long) COVID
	Effects of (long) COVID on day to day life
	Hospitals or Hospitalization
	Identity or Changes in identity
	Invisible illness or disability
	Mental health or Well-being
	Misdiagnosis
	Physical health
	Pre-existing conditions or Comorbidities
	Recovery from COVID or Failure to recover
	Related or suspected related health conditions (e.g., ME/CFS, POTS)
	Setbacks

	Signs or Symptoms (or lack thereof)
	Survival
	Turning points
**Resources**	Ability or inability to prove one has long COVID or a disability
	Access to healthcare
	Access to masks, vaccines, tests, treatments, etc.
	Accommodations (needed, requested, granted, not granted)
	Benefits (Health insurance / Disability, incl. claims/denials/appeals)
	Disadvantages or Disparities or Injustice
	Employment (including ability/inability to take time off of work or to return to work)
	Internet
	Money
	Time
**Social or Societal Factors**	Ableism
	Altruism
	Authoritarianism
	Belief or lack of belief in COVID, efficacy or safety of vaccines, etc.
	Caring or Empathy or Compassion (or lack thereof)
	Comparing oneself with others (e.g., I don't have it as bad)
	Desire to connect with others or Camaraderie
	Discrimination
	Doctor-patient relationship and communication
	Feeling blamed
	Feeling othered, misunderstood, disrespected, dismissed, degraded
	Gaslighting
	Helping, Protecting, or Advocating for oneself or others
	Isolation or Ostracization
	Not being or feeling listened to, understood, or believed
	Perceptions of sufficiency or efficacy of HCPs knowledge, care, advice, commitment, etc.
	Politics or politicization
	Self Interest
	Social support
	Stigma

	Trust or Mistrust in other people
	Validation or invalidation
	Voices (e.g., whose are heard vs. whose are not)

**Supplemental Table 2. Codes reflecting dimensions and abilities of health care access**<br>Data were coded using each code, described below. Key ideas were used to facilitate coding.		
**Code**	**Description**	**Key ideas**
Approachability	How visible and readily accessible healthcare services are for the people most in need of those services and who recognize they have a need	Perception of healthcare, society, visibility
Ability to perceive	The notion of ability to perceive need for care among populations is crucial and determined by factors such as health literacy, knowledge about health and beliefs related to health and sickness.	Awareness and understanding of Long COVID as medical issue
Acceptability	Reflects how much people accept the aspects of the healthcare services within the context of surrounding cultural and social factors, beliefs in the systems of medicine, and perceived appropriateness to seek care	Cultural norms, health beliefs, biases
Ability to seek	Relates to the concepts of personal autonomy and capacity to choose to seek care, knowledge about health care options and individual rights that would determine expressing the intention to obtain health care. A good example would be female discrimination regarding the initiation of care or abuse and neglect discouraging ethnic minorities to seek care.	Perceived receptivity of care providers, awareness of options, understanding of role of medicine in symptom relief
Availability and accommodation	Refer to the physical existence of health resources, qualified providers, and varied service delivery modes, that can be reached in a timely manner	Time, distance, available hours or providers
Ability to reach	The notion of personal mobility and availability of transportation, occupational flexibility, and knowledge about health services that would enable one person to physically reach service providers. Restricted mobility of the aged and handicapped, or the inability of casual workers to be absent from work to consult medical providers would be examples of these	Travel, cars, time off of work, social capital to aid in traveling
Affordability	Reflects people's economic capacity to spend resources and time to access, and assume the cost of accessing, relevant healthcare services	Cost, insurance, opportunity costs
Ability to pay	The capacity to generate economic resources - through income, savings, borrowing or loans - to pay for health care services without catastrophic expenditure of resources required for basic necessities (e.g. sale of home).	Reflections on costs and tradeoffs
Appropriateness	Reflects how well the services people are provided meet their needs related to timeliness, healthcare provider expertise, and accurate assessments and treatments.	Receptivity, accurate assessments, diagnoses
Ability to Engage	Participation and involvement of the client in decision-making and treatment decisions, which is in turn strongly determined by capacity and motivation to participate in care and commit to its completion. This dimension is strongly related to the capacity to communicate as well as notions of health literacy, self-efficacy and self-management in addition to the importance of receiving care that is actually appropriate for the person, given its resources and skills.	Health literacy, self-efficacy
Codebook built from definitions and concepts from Levesque et al.:<br>Levesque, J.-F., Harris, M. F., & Russell, G. (2013). Patient-centred access to health care: conceptualising access at the interface of health systems and populations. *International Journal for Equity in Health*, *12*(1), 18. https://doi.org/10.1186/1475-9276-12-18		
